# Provision of special diets to children in public nurseries and kindergartens in Kraków (Poland)

**DOI:** 10.3389/fnut.2024.1341062

**Published:** 2024-03-08

**Authors:** Beata Piórecka, Agnieszka Kozioł-Kozakowska, Przemysław Holko, Iwona Kowalska-Bobko, Paweł Kawalec

**Affiliations:** ^1^Department of Nutrition and Drug Research, Faculty of Health Sciences, Institute of Public Health, Jagiellonian University Medical College, Kraków, Poland; ^2^Department of Pediatrics, Gastroenterology and Nutrition, Faculty of Medicine, Pediatric Institute, Jagiellonian University Medical College, Kraków, Poland; ^3^Faculty of Health Sciences, Institute of Public Health, Jagiellonian University Medical College, Kraków, Poland

**Keywords:** special diet, organization of meals, child nutrition, nursery, kindergarten, Poland

## Abstract

**Background:**

A specialized diet could be due to an allergy or other medical needs and also religious or cultural reasons. This study aimed to assess the availability and provision of special diets in kindergartens and nurseries financed by the Municipality of Kraków.

**Methods:**

This observational cross-sectional study was based on a diagnostic survey carried out using the Computer-Assisted Web Interview method and addressed to the managers of nurseries (*n* = 21) and kindergartens (*n* = 71) and, separately, to the parents of children attending these facilities (*n* = 1,096). Non-parametric tests were applied for an unadjusted comparison between children at nurseries and those at kindergartens.

**Results:**

Children with particular dietary requirements received special diet meals in 95.2% of nurseries and 60.5% of kindergartens. The availability of special diets was associated with the type of facility (*p* = 0.001), the number of children who ate in the facility (*p* = 0.032), and the daily cost of meals served to children (*p* = 0.009). The cost of meals was higher in kindergartens that offered special diets vs. those that did not offer such diets (*p* < 0.001). According to parents, 96.4% of the total number of children ate meals served in the facilities. In nurseries, 16.1% of children were on a special diet (as per the doctor’s recommendations in 11.7% of cases and according to parents’ own choice in 4.4%). In kindergartens, a special diet was served to 12.7% of children (doctor’s recommendations, 8.5%; parents’ own choice, 4.2%). The most common reason for using a special diet was food allergy (8.2% of children in nurseries and 5.8% of children in kindergartens). It was reported more often by the parents of children attending nurseries than by the parents of children attending kindergartens (8.0% vs. 4.2%, *p* = 0.007). The requirement for a special diet was found to be associated with the age of children (*p* < 0.033) and the use of oral treatment for chronic disease (*p* < 0.001).

**Conclusion:**

Providing special diets for children is better in nurseries than in kindergartens. Legal regulations are urgently needed to ensure equal access to adequate nutrition for all children with special dietary needs in childcare facilities.

## 1 Introduction

In the last decade, the number of children attending childcare facilities in Poland has increased significantly. In 2021, nearly a quarter of children under the age of three attended nurseries, which is 93.5% more than in 2015 (nearly 100,000 places more). At the same time, the number of children attending kindergartens has increased by 3.5%. Considering the large number of children in childcare, special attention should be given to nutrition in such institutions (1).

Child nutrition should always follow relevant recommendations, including quantitative and qualitative guidelines (2, 3). As children spend several hours a day in childcare and educational institutions, proper nutrition has a profound impact on their current and future health (4, 5). Moreover, it is one of the most important factors affecting their development and shaping their eating habits. In Poland, children attending kindergartens and nurseries receive up to four meals (breakfast, midday snack, lunch, and afternoon snack), depending on the length of stay in the facility. These meals should cover 70–75% of the recommended daily calorie intake (6). Therefore, to meet the dietary needs of a growing child, nutrition in childcare facilities should be handled by nutrition professionals. This may be challenging in the Polish setting, where nurseries and kindergartens often do not employ a dietitian and the cooking staff may not have sufficient knowledge to prepare appropriate meals (6). These limitations result from the lack of detailed regulations and the fact that nutritional standards are too general in Poland. As a consequence, the menus in childcare facilities are not always well balanced, and food products and portion sizes tend not to be adjusted to age (7–11).

Nurseries and kindergartens are required to provide meals to children during their stay in the facility. The director of the educational institution is responsible, if possible, for providing the child with a dietary meal. A special diet might be required in children with food allergy or intolerance, or with other medical needs. Moreover, it might be required for religious or cultural reasons or to cater for children with vegetarian or vegan preferences. All menus must include information about substances or products that cause allergies or intolerances (12). In Poland, parents inform the facility about the child’s health condition (based on a certificate from the physician) and special nutritional needs. However, special diets are not available in all institutions. There are no data in the literature on the provision of special diets to children who attend public nurseries (children aged 0–3 years) and kindergartens (children aged 3–7 years) in Poland. The aim of this study was to assess the availability and provision of special diets in public kindergartens and nurseries financed by the Municipality of Kraków.

## 2 Materials and methods

### 2.1 Study design and data collection

The study was based on a diagnostic survey conducted using the Computer-Assisted Web Interview method (CAWI) and addressed to the managers of nurseries and kindergartens as well as to the parents of children attending these facilities. The responses were collected on the server of Jagiellonian University Medical College as part of its survey system. The questionnaire for parents was validated in a pilot study with a group representing 10% of the target group size. A link to the questionnaire was sent to the managers of childcare institutions by the Department of Social Policy and Health of the City of Kraków. Subsequently, it was sent to the parents of children via an internal mail system or the institution’s newsletter.

**Figure 1:**
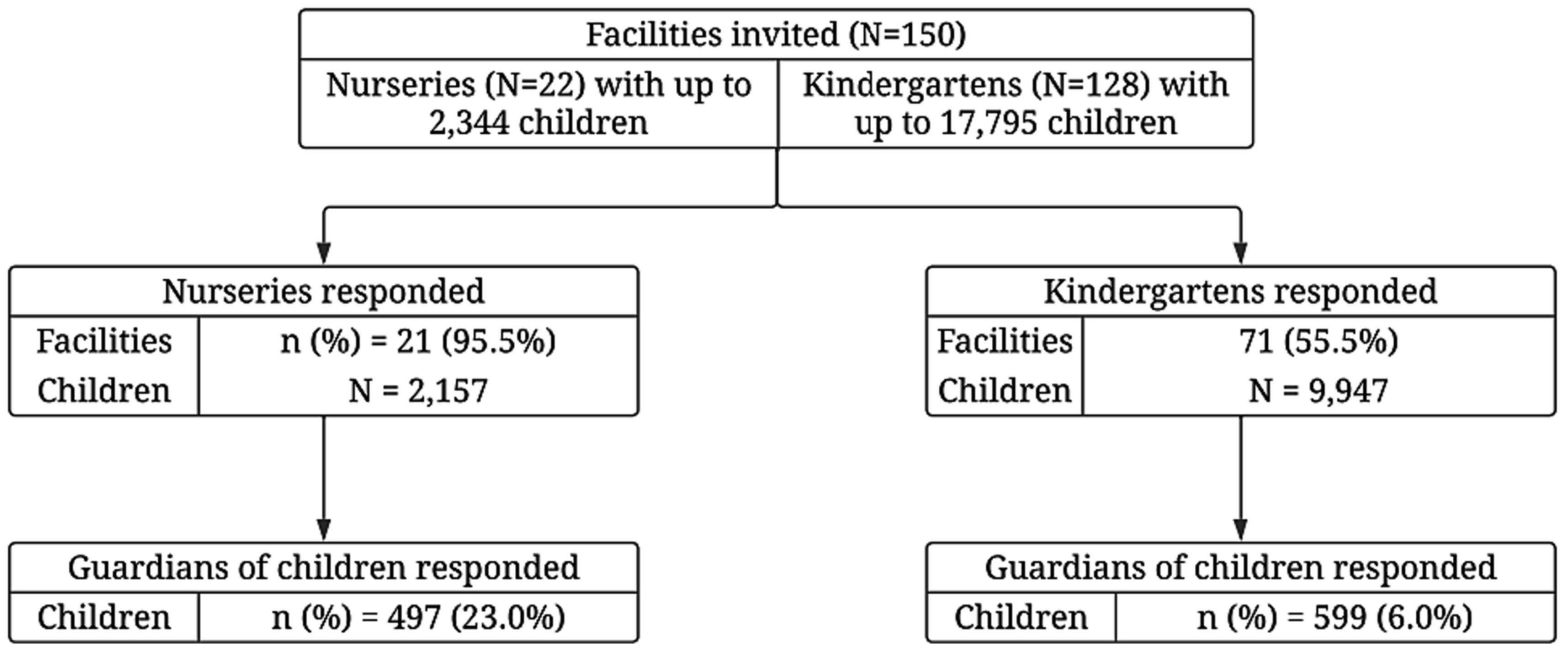
Flow diagram of the study.

In the 2021/2022 school year, there were 22 local community nurseries (2,344 places for children) and 128 kindergartens (17,795 places) funded by the City of Kraków (13). All participants who completed the questionnaire were enrolled in the study. The final study sample included 92 facilities and 1,096 parents (or legal guardians) of children (Figure 1).

The questionnaire for nurseries and kindergarten managers concerned the organization of meals and barriers to the provision of special diets. In addition, managers were asked about the number and cost of meals (including those for children with special nutritional needs), the kitchen staff and equipment organization, and measures taken to ensure food and nutrition safety. The questionnaire also contained questions about how meals offered in the facility are assessed and about the results of this assessment.

The questionnaire for parents also included questions related to the organization of meals and access to special diets in the facility. Additionally, questions regarding the assessment of nutrition provided in facilities and selected eating behaviors of children were included. In addition, it contained questions about the health status of children. Anthropometric data of the children – their weight and height – were collected from the parents. Using these data, the body mass index (BMI) was calculated and interpreted with national percentile charts (14), and the value was compared to BMI cutoff points provided by the International Obesity Task Force (the 85th percentile as overweight and 95th and more as obesity) (15).

The part of the study aimed at the parents of children was approved by the Jagiellonian University Bioethics Committee (No. 1072.6120.198.2022; as of August 31, 2022). The Helsinki University Ethics Review Board in Humanities and Social and Behavioural Sciences approved all procedures involving human subjects on 24 February 2015 (Statement 6/2015).

### 2.2 Statistical analysis

The results were presented as mean with standard deviation (SD) and median with interquartile range (IQR) for continuous variables or frequencies and percentages for categorical variables. The χ^2^ Pearson test for categorical variables and the Wilcoxon rank-sum test for continuous variables were applied for unadjusted comparisons between children attending nurseries and those attending kindergartens.

Generalized linear models (binomial distribution and logit link function) with robust variance estimators were used to assess differences between subgroups (i.e., nurseries vs. kindergartens, special meals vs. standard meals), controlling for all other variables that could affect the result (i.e., known characteristics of children, their parents, and/or childcare facility). The selection and assessment of the models were based on the Box–Cox test, the modified Park test, and the log-likelihood. Interactions between variables were included if their inclusion substantially improved the fit of the model to the data (i.e., >10% increase in log-likelihood) or if they resulted from the study design (e.g., age and facility type). Predictive margins were presented as adjusted means, while average marginal effects (for continuous variables) or contrasts of predictive margins (for categorical variables) were presented as adjusted differences in the results, with standard errors calculated using the delta method.

The analyses included all participants who completed the questionnaire (irrespective of the number of responses provided). Missing data were excluded from the analysis of an outcome. No multiplicity correction was implemented. A value of p of less than 0.05 was considered significant. Data were prepared and analyzed using Stata 17SE (StataCorp., College Station, TX, United States) and OriginPro 2021b (OriginLab Corporation, Northampton, MA, United States). The study was carried out according to the Strengthening the Reporting of Observational Studies in Epidemiology Statement (16).

## 3 Results

### 3.1 Relevant characteristics of facilities and study group

Among the facilities included in this study, all but one nursery had a kitchen where meals were prepared (in this one case the kitchen was shared with another facility). Out of 71 kindergartens, 67 had their kitchen, in 1 case the kitchen was rented, while external catering was provided only in 3 cases. Moreover, in all nurseries and kindergartens, the menus contained a list of allergens, and parents had access to the menu in the facility. In 96.5% of the nurseries and kindergartens, the kitchen staff was trained in healthy child nutrition and applicable regulations related to the organization of meals in educational institutions. All facilities reported that the nutrition staff assessed the menu’s compliance with current nutritional standards (energy and nutrient intake for a given age group). A dietitian was employed in 90.5% of nurseries and 12.7% of kindergartens. Among the facilities, 95.2% of nurseries and 60.6% of kindergartens offered meals for children with special nutritional needs (special diets) (Table 1).

**Table 1:** General characteristics of the nurseries and kindergartens participating in the study.

		Nurseries (*N* = 21)	Kindergartens (*N* = 71)	*p*-value
Number of children in the facility	Median (IQR), *N*	110.0 (94.0–116.0), 21	129.0 (100.0–155.0), 71	**0.003**
	Mean (*SD*), *N*	102.7 (25.6), 21	140.1 (65.5), 71	
Number of children with disabilities in the facility	Median (IQR), *N*	1.0 (0.0–4.0), 21	1.0 (0.0–4.0), 71	0.374
	Mean (*SD*), *N*	3.0 (3.7), 21	3.5 (7.1), 71	
Type of meal provision in the facility	Own kitchen: *n* (%), *N*	20 (95.2), 21	67 (94.4), 71	0.207
	Rented kitchen: *n* (%), *N*	0 (0.0), 21	1 (1.4), 71	
	External catering: *n* (%), *N*	0 (0.0), 21	3 (4.2), 71	
	Kitchen shared with other facility: *n* (%), *N*	1 (4.8), 21	0 (0.0), 71	
Number of children eating in the facility	Median (IQR), *N*	109.0 (62.0–115.0), 21	127.0 (100.0–153.0), 71	**0.001**
	Mean (*SD*), *N*	96.2 (30.2), 21	138.4 (65.3), 71	
Special diet meals offered in the facility		20 (95.2), 21	43 (60.6), 71	**0.003**
Number of children on a special diet in the facility	Median (IQR), *N*	8.0 (5.0–13.5), 20	5.0 (3.0–8.0), 43	**0.004**
	Mean (SD), *N*	9.7 (5.6), 20	5.7 (3.7), 43	
Daily cost of nutrition for a child on a special diet (PLN)	Median (IQR), *N*	6.5 (6.5–6.5), 18	10.0 (9.0–12.5), 38	**<0.001**
	Mean (*SD*), *N*	6.6 (0.3), 18	11.4 (2.9), 38	
Daily cost of nutrition for a child (PLN)	Median (IQR), *N*	6.5 (6.5–6.5), 21	10.0 (9.0–11.0), 71	**<0.001**
	Mean (*SD*), *N*	6.6 (0.3), 21	10.5 (2.0), 71	
A dietitian employed	*n* (%), *N*	19 (90.5), 21	9 (12.7), 71	**<0.001**
An assessment of children’s satisfaction with meals	*n* (%), *N*	5 (23.8), 21	45 (63.4), 71	**0.001**
Access–menu for parents provided	*n* (%), *N*	21 (100.0), 21	70 (98.6), 71	0.584
Free drinking water for children in the facility	*n* (%), *N*	18 (85.7), 21	68 (95.8), 71	0.101

Own kitchen, meals prepared for children in the nursery from scratch; external catering, meals are purchased from an external company; PLN1 (Polish Zloty) = €0.21 (as of September 2022). *p*-values that are statistically significant are highlighted in bold.

The questionnaire was completed by 1,096 parents of children attending nurseries and kindergartens. Almost all of them lived in Kraków (a city with more than 100,000 residents). Over 84% of mothers and 64% of fathers reported having a higher education degree. Most respondents (70.4%) described their financial situation as average. In the whole study group, 6.1% of children were overweight and 2.6% were obese (Table 2). Our results showed that about 96.5% of children ate meals served in the facility. The nutrition of the remaining children (*N* = 9) was based on individual external catering (11.1%) or meals prepared by parents (88.9%).

**Table 2:** General sociodemographic characteristics and nutritional status of the study group.

		Nurseries (*N* = 497)	Kindergartens (*N* = 599)	*p*-value
Sex: *n* (%), *N*	Girl	235 (47.3), 497	281 (46.9), 599	0.902
Age, years	Median (IQR), *N*	1.8 (1.4–2.3), 491	5.0 (4.0–6.0), 581	**<0.001**
	Mean (*SD*), *N*	1.8 (0.6), 491	5.4 (7.0), 581	
BMI, kg/m^2^	median (IQR), *N*	15.5 (14.5–16.6), 487	14.9 (13.9–15.9), 592	**<0.001**
	Mean (*SD*), *N*	15.6 (1.9), 487	15.1 (2.0), 592	
BMI category: *n* (%), *N*	Obesity	12 (2.5), 481	15 (2.6), 576	**<0.001**
	Overweight	42 (8.7), 481	23 (4.0), 576	
	Normal weight	372 (77.4), 481	416 (72.2), 576	
	Underweight	52 (11.4), 481	122 (21.2), 576	
Child on oral drugs for chronic disease	*n* (%), *N*	36 (7.2), 497	76 (12.7), 599	**0.003**
Child with a disability certificate	*n* (%), *N*	20 (4.0), 496	28 (4.7), 597	0.597
Child eating in the facility	*n* (%), *N*	479 (96.4), 497	578 (96.5), 599	0.918
Child’s physical activity (parent assessment): *n* (%), *N*	Low activity	4 (0.8), 497	13 (2.2), 598	**<0.001**
	Moderate activity	93 (18.7), 497	233 (39.0), 598	
	High activity	400 (80.5), 497	352 (58.9), 598	
Place of residence: *n* (%), *N*	Village	22 (4.4), 497	13 (2.2), 599	**0.034**
	City	475 (95.6), 497	586 (97.8), 599	
Number of persons in the household	Median (IQR), *N*	4.0 (3.0–4.0), 496	4.0 (3.0–4.0), 596	**<0.001**
	Mean (*SD*), *N*	3.7 (0.8), 496	4.0 (1.4), 596	
Number of underage persons in the household	Median (IQR), *N*	1.0 (1.0–2.0), 495	2.0 (1.0–2.0), 596	**<0.001**
	Mean (*SD*), *N*	1.4 (0.9), 495	1.7 (1.0), 596	
Parent with occupational activity	Father	62 (12.5), 497	96 (16.0), 599	**0.035**
	Mother	14 (2.8), 497	30 (5.0), 599	
	Both parents	421 (84.7), 497	473 (79.0), 599	
Self- assessed financial situation: *n* (%), *N*	Average	367 (73.8), 497	407 (67.9), 599	0.054
	Above average	104 (20.9), 497	163 (27.2), 599	
	Below average	26 (5.2), 497	29 (4.8), 599	
Education level: mother: *n* (%), *N*	Basic education	7 (1.4), 496	3 (0.5), 598	0.050
	Basic vocational education	4 (0.8), 496	16 (2.7), 598	
	Secondary education	62 (12.5), 496	79 (13.2), 598	
	Higher education	423 (85.3), 496	500 (83.6), 598	
Education level: father: *n* (%), *N*	Basic education	12 (2.4), 496	12 (2.0), 597	0.231
	Basic vocational education	24 (4.8), 496	43 (7.2), 597	
	Secondary education	148 (29.8), 496	155 (26.0), 597	
	Higher education	312 (62.9), 496	387 (64.8), 597	

BMI, body mass index, value less than 0.05 was considered significant chi-square test. *p*-values that are statistically significant are highlighted in bold.

### 3.2 Provision of special diets from the perspective of managers

The costs of meal provision were higher in kindergartens offering special diets compared with those that did not offer such diets (*p* < 0.001). The provision of special diet meals in a facility was associated with the type of facility (*p* = 0.001), the number of children who ate in the facility (*p* = 0.032), and the daily cost of meals served to children in the facility (*p* = 0.009) (Table 3).

**Table 3:** The association between the probability of providing special diet meals in a facility and selected facility characteristics.

		OR (95% CI)	*p*-value
Facility type	Nursery	Reference	
	Kindergarten	0.01 (0.0004–0.13)	0.001
Number of children with disabilities	Per 1 additional child	1.04 (0.92–1.19)	0.531
Number of children eating in the facility	Per 1 additional child	1.01 (1.00–1.02)	0.032
Daily cost of nutrition of a child	Per 1 PLN increase	1.65 (1.13–2.41)	0.009
A dietitian employed	No	Reference	
	Yes	1.00 (0.16–6.33)	0.996
Assessment of children’s satisfaction with meals	No	Reference	
	Yes	1.18 (0.38–3.67)	0.774

Logistic regression; *N* = 92; intercept = 0.27 (0.003–21.03).

After adjustment for other differences between nurseries and kindergartens, the probability that a nursery offered a special diet was nearly twice as high as that for a kindergarten [0.985 (95% CI: 0.956 to 1.000) vs. 0.497 (95% CI: 0.380 to 0.615)]. According to managers, the main reasons for the kitchen not being able to provide meals for children with special nutritional needs were as follows: no additional staff including a dietician (*n* = 19, 79.2%) and no additional space (*n* = 10, 41.7%).

### 3.3 Availability of special diets for children from the perspective of parents

**Figure 2:**
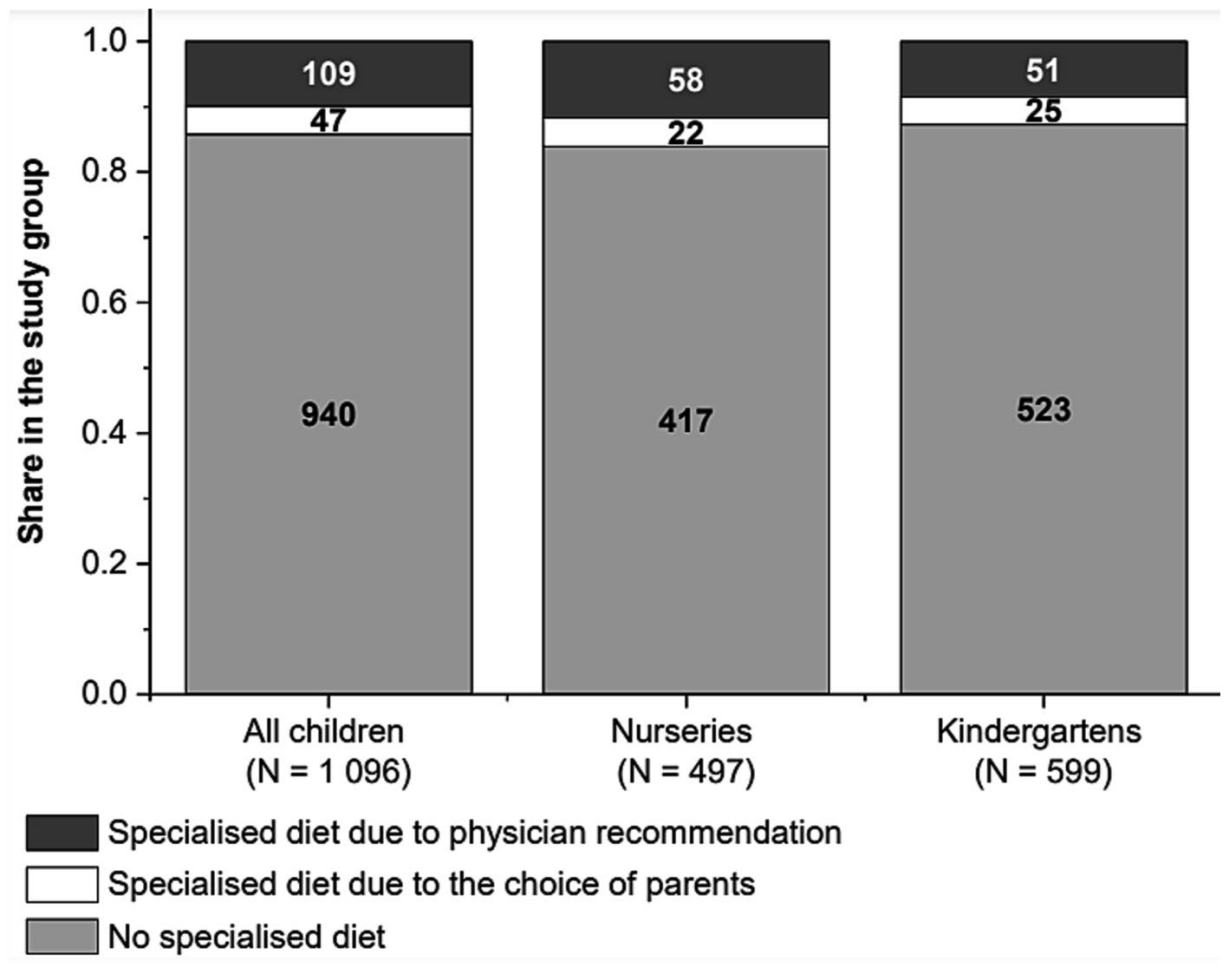
Frequency of children on a special diet in the whole study group and by childcare facility (nurseries vs. kindergartens: *p* = 0.209).

Overall, 14.2% of children had a special diet, more often due to the doctor’s recommendation than because of the parent’s request. In nurseries, 16.1% of children were on a special diet (as per the doctor’s recommendations in 11.7% of cases and according to parents’ own choice in 4.4%). In kindergartens, a special diet was served to 12.7% of children (doctor’s recommendations, 8.5%; parents’ own choice, 4.2%). Figure 2 shows the frequency of parents’ answers regarding the need to follow a diet in their child due to medical recommendations and due to the parents’ choice.

**Figure 3:**
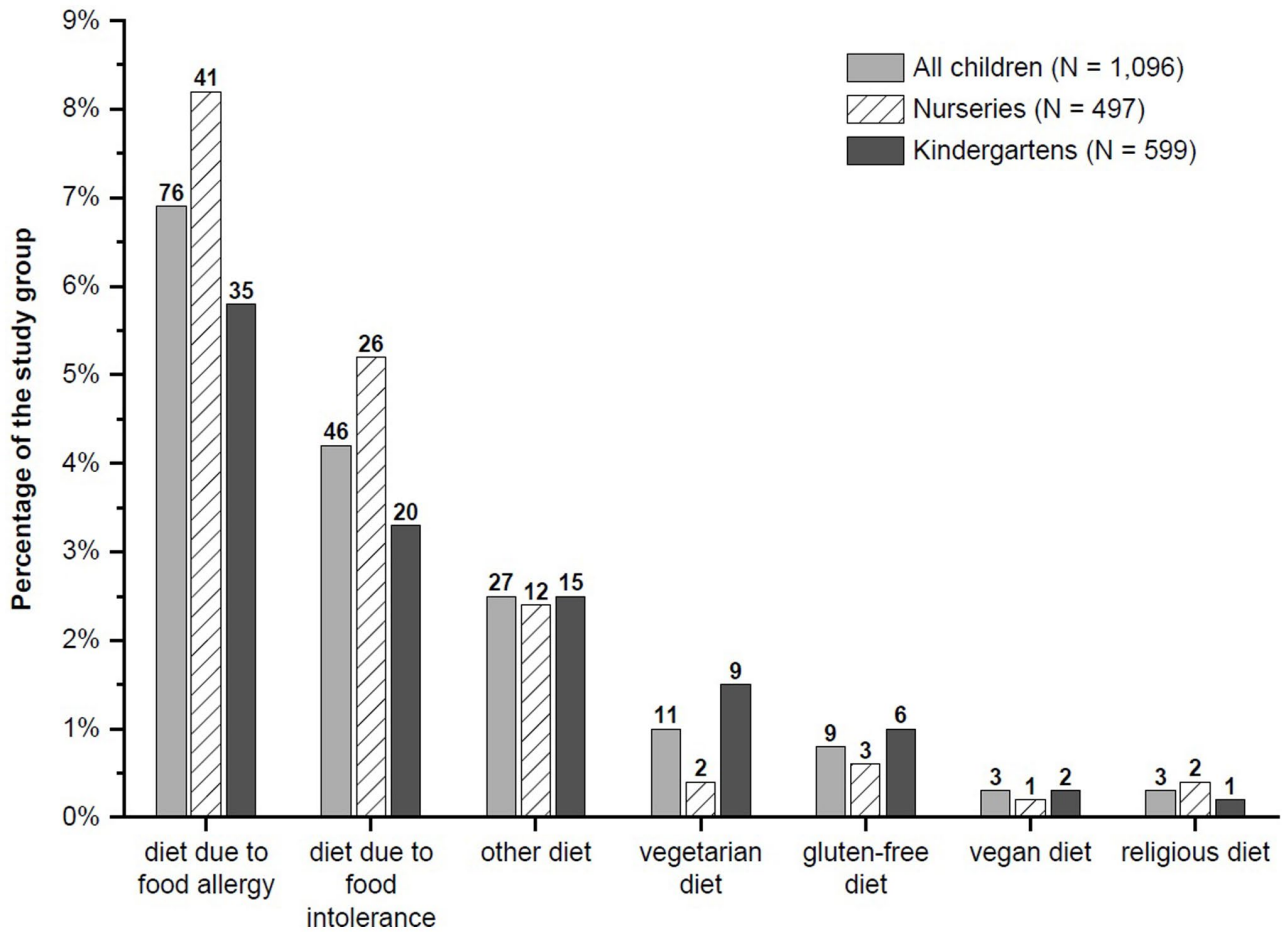
Percentage of children in the whole study group and by childcare facility, who received individual special diets as reported by parents. Nurseries vs. kindergartens: *p* > 0.05 for all comparisons (unadjusted for potential differences in other variables).

The most common reason for using a special diet in nurseries was food allergy (8.2% of all children). In kindergartens, the most common reasons were food allergy (5.8%) and food intolerance (3.3%). No weight loss diet was reported, but more than 1/4 of parents stated that their child requires a special diet for other reasons than those indicated in the survey (Figure 3).

Being on a special diet was associated with the age of children (directly and/or through the facility type in the whole study group and by the type of facility); a post-regression estimated value of *p* of (0.033) and the use of oral treatment for chronic disease (*p* < 0.001) (Supplementary Table S1).

**Figure 4:**
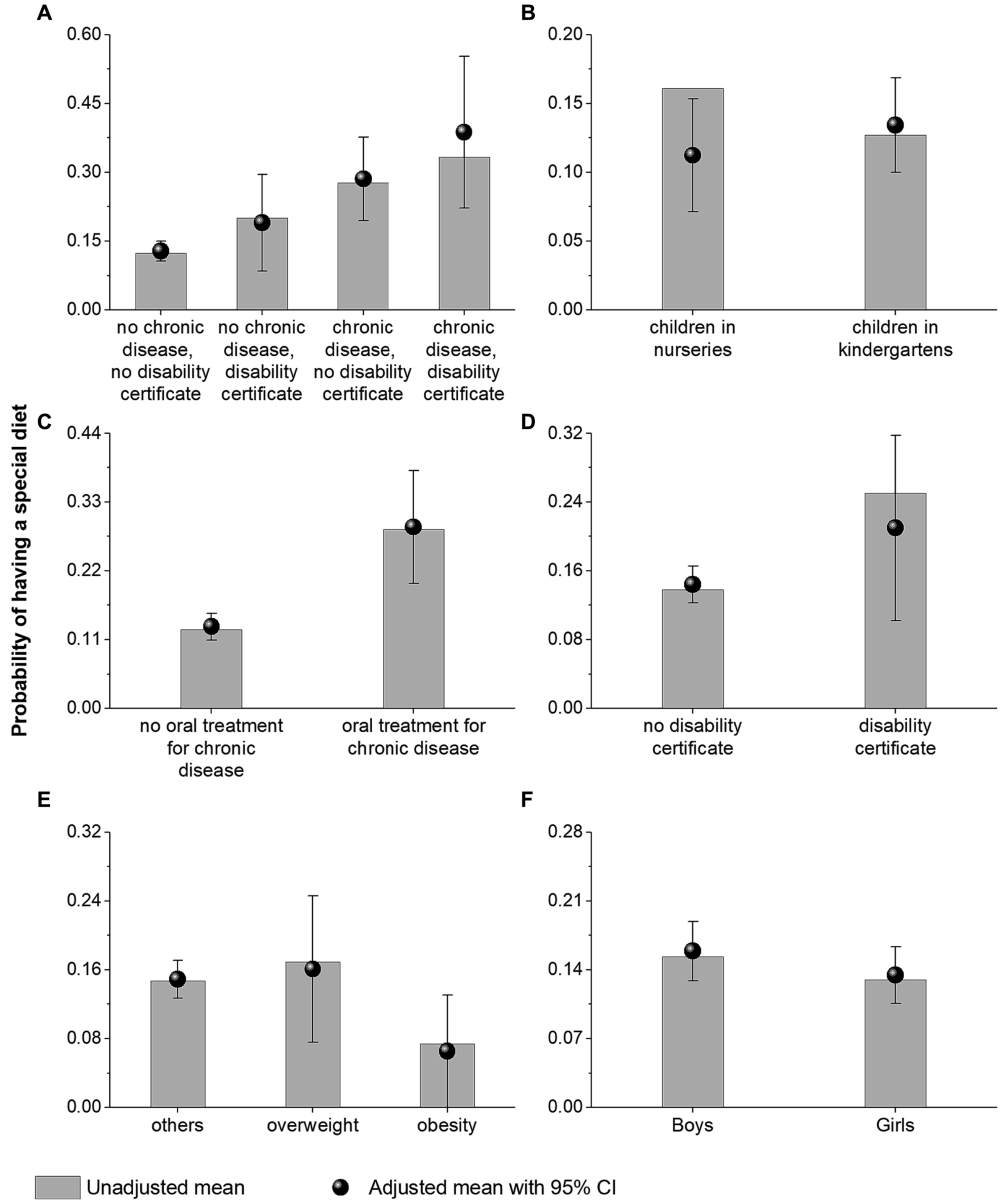
Adjusted and unadjusted (raw) probabilities of having a special diet among the subgroups of children depending on a disability certificate and chronic diseases **(A)**, type of childcare facility **(B)**, oral treatment for chronic diseases **(C)**, disability certificate **(D)**, body mass index category **(E)**, and sex **(F)**.

The association between the need for a special diet and the fact of having a disability certificate was not significant (*p* = 0.187; disability was reported for 4.4% of children). However, the adjusted probabilities of having a special diet in subgroups indicated that this aspect may have potential clinical importance. Children with a disability certificate more often were on a special diet than those without such a certificate. This applied particularly to the subgroup of children receiving oral treatment for chronic disease (Figure 4).

## 4 Discussion

The objective of the study was to assess the availability and provision of special diets in public kindergartens and nurseries financed by the Municipality of Kraków. The study was aimed at the managers of public nurseries and kindergartens, as well as the parents of children attending these facilities.

In Poland, there are no legal regulations that oblige education and childcare facilities to provide a special diet for children (17). According to the parents’ responses, 16.1% of children attending nurseries and 12.7% of those attending kindergartens followed a special diet. On the other hand, according to the managers, a special diet was provided to 9% of children in nurseries and 3% of children in kindergartens. This shows that not all children receive a special diet in the facility. The discrepancy in responses may be caused by the fact that some parents do not report the need for a special diet to the facility because they do not have a medical certificate from a physician. In Poland, the decision whether to recognize the child’s need for a special diet lies at the discretion of the facility managers. This is because there are no uniform regulations to provide adequate guidance, as well as kitchens and their employees are not prepared to serve meals to children with special nutritional needs.

The situation in Poland is different from that in other European countries. In Sweden, for example, parents report the need for a special diet in the facility. In a cross-sectional study by Servin et al. (18), 19% of preschool children were on special diets, both for medical (6.3%) and non-medical reasons (12%). Almost half of the children (47%) on special diets had no medical certificate. The authors concluded that mandatory medical certificates for special diets due to medical conditions could reduce unnecessary dietary restrictions in children.

Lyons et al. (19) studied the prevalence of food sensitization and food allergy in children across Europe. In Poland, the prevalence of self-reported food allergy was 24.6%, which is higher than that observed in our study (8.4 and 5.9% of children in nurseries and kindergartens, respectively). Therefore, the number of children with special nutritional needs is likely to increase in the coming years, presenting a new challenge for childcare facilities.

In our study, a special diet was required more often for medical reasons, such as food allergies and intolerance. A much lower percentage of children had special dietary needs due to family preferences or beliefs (veganism or vegetarianism). More than 1/4 of parents stated that their child requires a special diet for other reasons than those indicated in the survey. We did not collect possible other needs for a specialized diet in a child. The percentage of children on a gluten-free diet was surprisingly low, considering that this diet is becoming increasingly popular and is often used without medical indications (20).

In a Swedish survey of 3,221 children aged 1–5 years, the five most common diets were pork-free (7.8%), vegetarian (4.8%), cow’s milk-free (3.5%), egg-free (1.2%), and lactose-free (1.1%) diets (18). The most common food allergens in the group of preschool children from Lublin (Poland) were cow’s milk proteins, nuts, egg whites, small seeds, cocoa, celery, and citrus fruits (21).

To provide a special diet, the personnel at the facility should have adequate qualifications. However, in Poland, educational facilities have no legal obligation to hire a dietitian or to train the staff in the principles of nutrition and nutrition planning. Meals are usually planned by staff without expertise in this area, which is confirmed by other studies (6, 22). This translates not only to the inability to serve special diets but also to the poor quality of meals provided in the facilities. Although the managers of the facilities reported that their staff were trained in child nutrition, their qualifications were still insufficient to prepare special meals. This was reported by the managers as the most important barrier to providing a special diet.

The results of our study showed that most facilities had their own kitchen, with only a small percentage of nurseries and kindergartens using external catering services. This is in line with a study by Myszkowska-Ryciak and Harton (23), who also reported that some of the facilities opted for catering instead of their own kitchen. When running the kitchen in childcare facilities, the management of the food safety system and the identification of corrective actions are necessary to eliminate any risks associated with preparing meals. In our study, the HACCP systems were implemented in all nurseries and most kindergartens. Trafiałek et al. (24) reported that nurseries in Warsaw declared a high level of compliance with both the GMP/GHP and HACCP standards in terms of documentation, but in practice, compliance was much lower, especially for the HACCP system.

A sanitary report on inspections in Polish educational system units (excluding nurseries) in the school year 2020/2021 revealed numerous irregularities. These included inadequate technical or sanitary conditions in the kitchen, deficiencies in kitchen equipment, lack of systematic supplementation of GHP/GMP and/or HACCP system documentation, and no information on nutrient composition of the prepared meals, including ingredients that cause allergies or intolerances (25).

In recent years, little has changed in the field of nutrition in childcare facilities in Poland. Research conducted in 2016 showed that public facilities had a much lower nutritional rate. Moreover, most of the facilities had their own kitchen and did not employ a dietitian to plan the meals, especially for children with special dietary requirements. Some errors were also observed in child menu planning. The authors emphasized the lack of uniform recommendations on nutrition for children in nurseries that would be mandatory as well as easy to understand and implement (23). Seven years later, our study showed that there has been a considerable improvement in this area, especially in nurseries. A dietitian is employed in most nurseries, and there is a better availability of special diets. However, little progress has been shown for public kindergartens, with only less than 20% employing a dietitian. As mentioned above, this continues to be the most important barrier to the provision of special diets in kindergartens. Our study also suggests that the higher cost of a special diet may be an additional barrier. This constitutes a significant challenge, considering that the costs of meal provision in childcare and educational institutions in Poland are generally not reimbursed. Meanwhile, research shows that reimbursement rates were positively associated with food expenditures and the nutritional quality of food served in the institutions and could improve child nutrition (26).

Recently, much attention has been paid to the growing problem of obesity among children (27). Nutritional errors are the main cause of obesity, and the food that children receive in institutions has a significant impact on the development of this disease. In Poland, the highest prevalence of obesity is observed among the youngest children, that is, the age group of up to 36 months. Overweight and obesity affects about 10% of children up to 3 years of age and 30% of children in early school age (28, 29). In our study, 8.7% of children were classified as overweight or obese. However, these calculations were based on values provided by parents, which may lead to underestimation. The problem of obesity requires special attention, both in terms of treatment and prevention and this is also the responsibility of an educational institution. It is recommended that children receive mainly water to drink and that it is easily accessible (30, 31). In our study, children had access to water at most facilities where it was usually available in playrooms. Water dispensers in the facilities were much less common. However, research shows that although there is increased availability of water, which should be considered a beneficial and health-promoting change, the menus in nurseries and kindergartens are still dominated by other beverages, mainly tea with added sugar or honey (32, 33).

According to Polish law, facilities must comply only with recommendations for the implementation of nutritional standards (2). However, these standards do not specify the range of products but only the calorie and nutrient content in the diet. Recommendations on the regulation of nutrition principles in educational facilities concerning portion size, food quality, and eating time have been included in a recent position statement on childhood obesity in Poland (34).

### 4.1 Strengths and limitations of the study

Our study has strengths and limitations. To our knowledge, this is the first study on the provision of special diets to children with special dietary needs in childcare institutions. One potential limitation of the study is that it collected self-reported data, and thus some information might have been omitted, particularly where facility managers were involved. This may have implications for the general representation of the facilities under review. The online character of the study is another limitation. As better-educated people use the Internet more often, the representativeness of the group regarding the level of education is reduced. Another reason for the lower representativeness is the fact that people with higher education are more interested in health-related issues (35). In our study, more than 80% of the participants had a higher education degree, which does not correspond to the distribution of the level of education in the entire population. Furthermore, the generalizability of this study may be limited by the 55.5% response rate among kindergarten managers. In many studies conducted over the Internet, the tendency to participate in the survey is low, mainly due to the lack of time, but also because it is easy to ignore the invitation.

## 5 Conclusion

A well-balanced diet in institutions should be available to all children, including those who have special dietary requirements. The study showed that most of the nurseries and more than half of the kindergartens provided meals for children with special nutritional needs. However, the results indicate that more children require a special diet than the number of children who actually receive such a diet in the facility. In kindergartens, the main barrier to preparing meals for children with special nutritional needs was the lack of dietitian support. Therefore, legal regulations are urgently needed to ensure equal access to adequate nutrition for all children. One of the first actions to be undertaken is to introduce a requirement for nurseries and kindergartens to employ a dietitian. Consequently, the managers of childcare facilities may need more economic resources to implement the provision of special diet meals to children. Finally, there is a need to improve the knowledge and competence about nutrition both among the personnel (mainly in kindergartens) and among parents themselves (especially the parents of children in nurseries).

## Data availability statement

The raw data supporting the conclusions of this article will be made available by the authors, without undue reservation.

## Ethics statement

The studies involving humans were approved by the Jagiellonian University Bioethics Committee (No. 1072.6120.198.2022; as of August 31, 2022). The Helsinki University Ethics Review Board in Humanities and Social and Behavioral Sciences approved all procedures involving human subjects on 24 February 2015 (Statement 6/2015). The studies were conducted in accordance with the local legislation and institutional requirements. The participants provided their written informed consent to participate in this study.

## Author contributions

BP: Conceptualization, Data curation, Formal analysis, Investigation, Methodology, Resources, Writing – original draft, Writing – review & editing, Project administration. AK-K: Conceptualization, Data curation, Formal analysis, Investigation, Methodology, Writing – original draft. PH: Data curation, Formal analysis, Software, Visualization, Writing – original draft. IK-B: Funding acquisition, Project administration, Supervision, Writing – review & editing. PK: Data curation, Formal analysis, Funding acquisition, Supervision, Validation, Writing – review & editing.
