# Supplementary material for: Provision of special diets to children in public nurseries and kindergartens in Kraków (Poland)
Source: Front Nutr. 2024 Mar 8;11:1341062. doi: 10.3389/fnut.2024.1341062 (PMC10957766; doi:10.3389/fnut.2024.1341062)
Supplement: Supplementary file 1 [file Table_1.docx]

Supplementary Table 1. The association between the probability of having a special diet and the characteristics of children and their parents.

| Characteristic |  | OR (95% CI) | p-value |
| --- | --- | --- | --- |
| Facility type | Nursery | *Reference* |  |
|  | Kindergarten | 0.43 (0.16 to 1.15) | 0.093 |
| Children age | Per 1 year increase | 0.68 (0.46 to 0.99) | 0.046 |
| Interaction of facility type and age | Nursery # no age change | *Reference* |  |
|  | Kindergarten # 1-year increase of age | 1.41 (0.94 to 2.12) | 0.095 |
| Sex | Boy | *Reference* |  |
|  | Girl | 0.81 (0.57 to 1.16) | 0.250 |
| BMI category | Normal or underweight (others) | *Reference* |  |
|  | Overweight | 1.10 (0.55 to 2.19) | 0.787 |
|  | Obesity | 0.38 (0.09 to 1.61) | 0.190 |
| Oral treatment for chronic disease | No | *Reference* |  |
|  | Yes | 2.82 (1.71 to 4.66) | <0.001 |
| Disability certificate | No | *Reference* |  |
|  | Yes | 1.62 (0.79 to 3.32) | 0.187 |
| Physical activity | Low | *Reference* |  |
|  | Moderate | 0.58 (0.17 to 1.94) | 0.374 |
|  | High | 0.69 (0.21 to 2.23) | 0.535 |
| Place of residence | Village | *Reference* |  |
|  | City | 5.37 (0.76 to 37.78) | 0.091 |
| Number of persons in the household | Per 1 additional person | 1.09 (0.97 to 1.24) | 0.147 |
| Parents with occupational activity | Father | *Reference* |  |
|  | Mother | 2.31 (0.95 to 5.65) | 0.065 |
|  | Both parents | 0.82 (0.50 to 1.34) | 0.435 |
| Self- assessed financial situation | Average | *Reference* |  |
|  | Above average | 0.91 (0.59 to 1.41) | 0.675 |
|  | Below average | 0.70 (0.27 to 1.82) | 0.465 |
| Education level: mother | Basic education | *Reference* |  |
|  | Basic vocational education | 0.45 (0.02 to 8.94) | 0.600 |
|  | Secondary education | 0.64 (0.09 to 4.80) | 0.667 |
|  | Higher education | 1.10 (0.14 to 8.53) | 0.928 |
| Education level: father | Basic education | *Reference* |  |
|  | Basic vocational education | 1.25 (0.19 to 8.10) | 0.817 |
|  | Secondary education | 1.61 (0.24 to 10.68) | 0.620 |
|  | Higher education | 1.54 (0.22 to 10.61) | 0.661 |
